# Comparison of Guide to Expression of Uncertainty in Measurement and Monte Carlo Method for Evaluating Gauge Factor Calibration Test Uncertainty of High-Temperature Wire Strain Gauge

**DOI:** 10.3390/s25051633

**Published:** 2025-03-06

**Authors:** Yazhi Zhao, Fengling Zhang, Yanting Ai, Jing Tian, Zhi Wang

**Affiliations:** Liaoning Key Laboratory of Advanced Measurement and Test Technology for Aviation Propulsion System, School of Aero-Engine, Shenyang Aerospace University, Shenyang 110136, China; zhaoyazhi@stu.sau.edu.cn (Y.Z.); ytai@163.com (Y.A.); tianjing@188.com (J.T.); wangzi629@163.com (Z.W.)

**Keywords:** gauge factor, calibration test, GUM, Monte Carlo method, uncertainty

## Abstract

High-temperature strain gauges are widely used in the strain monitoring of the hot-end components of aero-engines. In the application of strain gauges, the calibration of the gauge factor (GF) is the most critical link. Evaluating the uncertainty of GF is of great significance to the accuracy analysis of measurement results. Firstly, the calibration test of the GF of the Pt-W high-temperature strain gauge was carried out in the range of 25 °C to 900 °C. The real test data required for the uncertainty evaluation were obtained. Secondly, the guide to the expression of uncertainty in measurement (GUM) and the Monte Carlo method (MCM) were used to evaluate the uncertainty of GF calibration test. The evaluation results of GUM and MCM were compared. Finally, the concept of the weight coefficient W was proposed to quantitatively analyze the influence of each input on the uncertainty of the output GF. The main uncertainty source was found, which had important engineering practical significance. The results show that the mean value of GF decreases with the increase in temperature nonlinearly. At 25 °C, GF is 3.29, and at 900 °C, GF decreases to 1.6. Through comparison and verification, the uncertainty interval given by MCM is closer to the real situation. MCM is superior to GUM, which only uses prior information for uncertainty assessment. MCM is more suitable for evaluating GF uncertainty. Among multiple uncertain sources, the weight coefficient W can effectively analyze Δε as the main uncertain source.

## 1. Introduction

Aero-engines are complex and precision thermodynamic machines. To ensure the normal operation of engines in extreme working environments such as high temperature, high pressure, and strong vibration, it is necessary to monitor the stress and strain generated by the hot-end components timely, accurately, and stably [1,2]. Therefore, convenient, effective high-temperature resistance strain gauges with high precision have emerged. In the development and application of high-temperature strain gauges, GF calibration is the most crucial link [3,4]. Measurement uncertainty is an important parameter to characterize calibration quality.

In the calibration test of high-temperature strain gauges, GF is affected by various physical factors such as the fabrication, installation, and testing of the strain gauges [5,6,7]. Therefore, there are many uncertainty factors in the GF calibration test. Uncertainty evaluation is a key step in the test work, without which the scientific conclusion cannot be drawn. Due to the complexity of the environment where strain gauges are located, the quality of strain measurement data has always been a concern in the aerospace field, but there is no unified evaluation standard [8]. Currently, through traditional statistical methods, the mean and variance of GF can be obtained, and then the dispersion interval can be obtained. However, there are few studies on the evaluation of the uncertainty of GF calibration tests. There are two main methods for evaluating measurement uncertainty. One is the “Uncertainty of Measurement—part 3: Guide to the Expression of Uncertainty in Measurement (2010)” (GUM) based on the law of propagation of uncertainty [9]. This method is the most widely used and easy to operate, but it is mainly applicable to linear models and has certain limitations [10]. Hayat El Fazani et al. conducted tensile tests on additive manufacturing specimens and analyzed the uncertainty of the specimen’s performance with GUM [11]. Yingming Chen et al. evaluated the monitoring uncertainty of the high-speed rail long-wave timing system based on GUM [12]. Song Mingshun et al. used Bayes’ theorem and GUM to assess the measurement uncertainty of small samples. By comparison, Bayes’ theorem was more reliable than GUM [13].

Another uncertainty assessment method is the “Evaluation of Measurement Data—Supplement 1 to the Guide to the Expression of Uncertainty in Measurement—Propagation of Distributions Using a Monte Carlo Method (2008)” (Monte Carlo method, MCM) [14,15]. Jiang Wensong et al. used MCM to establish an evaluation model for the parameter calibration uncertainty of a 6-DOF manipulator. Through a large number of random numerical simulations, the uncertainty evaluation of the manipulator calibration was realized [16]. Huang Meifa et al. used MCM and an improved quasi-MCM to simulate and assess the uncertainty of cylindrical volume measurement and compared the results [17]. Jasveer Singh et al. used MCM to evaluate the uncertainty of the effective area of the pressure balancer and analyzed the influence of the input probability density function (PDF) on the output PDF [18]. Marius Forster applied MCM to the estimation of the local measurement uncertainty in transient heat-transfer experiments, demonstrating the advantages of MCM while considering nonlinearity and local uncertainties [19]. Kerstin Rost evaluated the measurement uncertainty of the gear measuring instrument by MCM and extended the mathematical model during the measurement process [20]. H.B. Motra applied MCM to evaluate the measurement uncertainty of material property and provided mathematical and calculation tools for the quantitative assessment of the quality of measurement results [21].

However, it is difficult to determine whether the above two methods are applicable in specific fields. Many scholars have compared and verified GUM and MCM in their respective fields. Saeid Sepahi-Boroujeni evaluated the uncertainty of the internal detection point set of the five-axis machine tool based on GUM and compared it with the MCM evaluation result [22]. Wang Wei et al. presented the principle and procedure of evaluating the measurement uncertainty of complex models by MCM and GUM and compared the two assessment methods with examples [23]. H.F.F. Castro introduced a method for calibrating small verifiers using the weighing method, evaluated the uncertainty of verification by GUM and MCM, respectively, and verified the validity of GUM by MCM [24]. Andrew Chen developed a sweat measurement system in the biomedical field and evaluated and compared the measurement uncertainty with GUM and MCM. The assessment result of GUM provided useful information for improving the measurement performance, while MCM could not evaluate it [25].

At present, in the calibration test of GF, the output GF is analyzed by traditional statistical method [26]. The high-temperature strain gauges are expensive, and the number of samples obtained by the test is small. The dispersion obtained by the existing statistical method cannot represent the overall characteristics, which directly affects the analysis accuracy of the test results and cannot reflect the influence of the input on the dispersion. Uncertainty is an important indicator to characterize the quality of measurement results, but there are few studies on the uncertainty evaluation of GF calibration tests.

In order to solve the above problems and find a more suitable method for GF uncertainty evaluation, this study was carried out. In this paper, the calibration test of the high-temperature strain gauge GF was carried out in the range of room temperature to 900 °C, and the real test data were obtained. The mathematical model was established. The GF uncertainty of strain gauge was evaluated by GUM and MCM methods, respectively. The MCM is based on the theorem of large numbers and is used to verify the GUM evaluation results. The results show that GUM method is not suitable for evaluating the uncertainty of GF calibration test of strain gauge. The concept of weight coefficient W was proposed, which can quantitatively analyze the influence of each input on the uncertainty of GF. It is a good supplement to MCM and has important practical significance in engineering.

## 2. Mathematical Model

**Figure 1:**
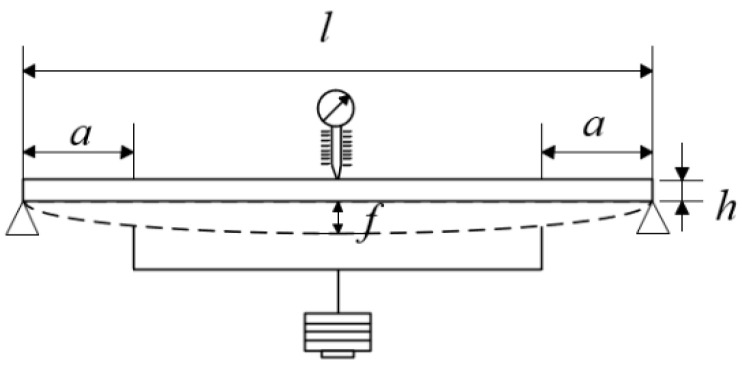
*GF* calibration test device schematic.

In the calibration test of the high-temperature strain gauge, plasma-sprayed ceramic Al_2_O_3_ is used to install the strain gauge on the specimen and connected to the strain meter. The specimen and support are placed in the high-temperature furnace, and heated step by step according to a certain temperature rise rate and temperature interval. Heat preservation is measured at each calibration temperature point, and then loading and unloading are assessed three times [27]. The data are recorded by external trigger mode. The schematic diagram of the GF calibration test of the high-temperature strain gauge is shown in Figure 1.

According to Hooke’s law, the surface strain $\varepsilon_{l/2}$ at the midpoint of the specimen can be calculated by measuring the midpoint deflection, as shown in Equation (1):

(1) $$\varepsilon_{l/2}=\frac{12h}{3l^{2}-4a^{2}}f_{l/2}\times 10^{6}$$

where h is the thickness of the specimen. l is the distance between the two fulcrums. a is the distance between the loading point and the fulcrum. $f_{l/2}$ is the difference in deflection between loading and unloading.

In order to avoid the error caused by direct measurement of resistance changes, a strain meter is used for strain measurement. The GF of a single strain gauge can be calculated from Equation (2):

(2) $$GF=\frac{GF_{0}\cdot \Delta \varepsilon }{\varepsilon_{l/2}}$$

where GF is the gauge factor that needs to be measured and calibrated. $GF_{0}$ is the initial gauge factor of the resistance strain meter, usually $GF_{0}=2$. Δε is the difference between the indication value of the strain meter during loading and unloading. The mathematical model of the calibration test is obtained by substituting Equation (1) into Equation (2), namely Equation (3):

(3) $$GF=\frac{\Delta \varepsilon (3l^{2}-4a^{2})}{6hf_{l/2}\times 10^{6}}=\frac{2\Delta \varepsilon }{\varepsilon_{l/2}}$$

## 3. Evaluation Principle

At present, in the field of measurement, GUM and MCM are the main methods for evaluating measurement uncertainty. However, in the calibration test of strain gauge *GF*, it is difficult to judge whether the two methods are applicable, and the evaluation results need to be analyzed and compared.

### 3.1. GUM Evaluation Principle

#### 3.1.1. Calculate the Standard Uncertainty

According to the measurement experience, the uncertainty evaluation methods of each input are classified into two categories: A and B. Category A is for quantities that are measured repeatedly, and Class B is for empirical probabilities. Δε is evaluated according to Class A. In the calibration test, there are six strain gauges attached to the upper surface of the specimen. In the heat preservation process of each temperature point, loading and unloading are carried out three times [28,29], and the 18 measured values of the input quantities Δε are obtained. $\bar{\Delta \varepsilon }$ represents the mean value of Δε, and the standard deviation $s_{p}\left(\Delta \varepsilon \right)$ of Δε can be calculated by Equation (4):

(4) $$s_{p}\left(\Delta \varepsilon \right)=\sqrt{\frac{1}{N\times (K-1)}\overset{N}{\underset{n=1}{\sum }}\overset{K}{\underset{k=1}{\sum }}{\left(\Delta \varepsilon_{nk}-\bar{\Delta \varepsilon_{n}}\right)}^{2}}$$

where N is the number of strain gauges, N=6. K is the number of loading and unloading times, K=3.

The standard uncertainty of Δε is calculated according to Equation (5) [9]:

(5) $$u\left(\bar{\Delta \varepsilon }\right)=\frac{s_{p}\left(\Delta \varepsilon \right)}{\sqrt{N\times K}}=\frac{s_{p}\left(\Delta \varepsilon \right)}{\sqrt{18}}$$

The uncertainty of h, l, and a in the mathematical model are calculated according to Class B assessment. The standard uncertainties of the input quantities are calculated according to Equation (6):

(6) $$u\left(x\right)=\frac{\gamma }{C}$$

where γ is the half width of the interval. C is the inclusion factor.

Generally, at room temperature, the uncertainty resulting from the maximum allowable error of a measuring instrument, usually assumed to be uniformly distributed [9]. γ(h)=0.02 mm, γ(a)=γ(l)=0.5 mm, and $C=\sqrt{3}$ is taken. With the increase in temperature, when the measured value is affected by a variety of random quantities, it is approximately a normal distribution, C=1.96.

In the test, the deflection is measured three times. The number of measurements is small. The standard uncertainty of f is calculated by the range method and is obtained from Equation (7):

(7) $$u\left(f\right)=\frac{s(f)}{\sqrt{K}}=\frac{s(f)}{\sqrt{3}}$$

where K is the number of loading and unloading times, K=3.

#### 3.1.2. Calculate the Combined Standard Uncertainty

According to the mathematical model Equation (3), input Δε and $\varepsilon_{l/2}$ are independent of each other. The combined standard uncertainty $u_{c}(GF)$ of the gauge factor is calculated by Equation (8):

(8) $$u_{c}\left(GF\right)=\sqrt{{\left[\frac{\partial GF}{\partial \Delta \varepsilon }\right]}^{2}u^{2}\left(\Delta \varepsilon \right)+{\left[\frac{\partial GF}{\partial \varepsilon_{l/2}}\right]}^{2}u^{2}\left(\varepsilon_{l/2}\right)}$$

Calculate the expanded uncertainty

(9) $$U(GF)=mu_{c}$$

where m is the coverage factor. In the usual measurement, m=2 is generally taken. When taking other values, the source should be stated [9].

### 3.2. MCM Evaluation and Verification Principle

#### 3.2.1. MCM Evaluation Principle

The numerical characteristics of input quantities are calculated from the obtained real test data. In the MATLAB R2021a software, *rand*() can generate uniformly distributed random numbers. The generator *normrnd*() can generate normally distributed gaussian numbers. At 25 °C, the uncertainties of the input quantities h, l, and a are determined by the maximum allowable error of the measuring instrument, which is assumed to follow a uniform distribution. The input quantities f and Δε are measured using dial indicator and strain gauges, and are assumed to follow gaussian distribution. As the temperature rises, h, l, and a are affected by many random factors, such as length, temperature difference, thermal expansion coefficient, etc. [30,31]. Therefore, it is assumed to follow a normal distribution.

**Figure 2:**
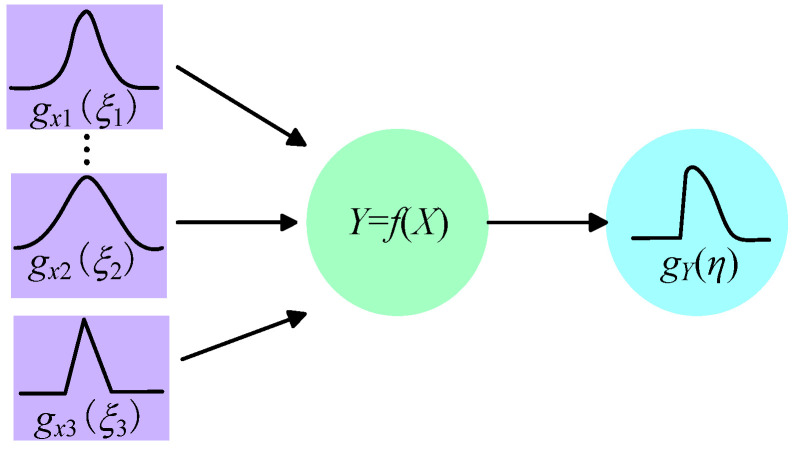
Schematic diagram of MCM distribution propagation.

The principle of MCM is the PDF of each input is propagated through the model to obtain the PDF of the output GF, as shown in Figure 2. The estimated value, the standard uncertainty, and the coverage interval of GF are obtained by further calculation.

The sample size M of the input should satisfy $M\geq [1/\left(1-p\right)]\times 10^{4}$, where p is the coverage probability. When p=95%, $M\geq 2\times 10^{5}$, and the sample size $M=10^{6}$ is taken.

#### 3.2.2. Verifying GUM by MCM

Although GUM is suitable for many situations, it is very difficult to determine whether it satisfies the applied environment. Because the basis of MCM is the large numbers theorem of probability theory and the application scope is wider, MCM is used to verify GUM. The endpoint deviation values $d_{\mathrm{low}}$ and $d_{\mathrm{high}}$ are calculated, respectively, according to Equations (10) and (11) [14]:

(10) $$d_{\mathrm{low}}=\left|y-U_{p}-y_{\mathrm{low}}\right|$$

(11) $$d_{\mathrm{high}}=\left|y+U_{p}-y_{\mathrm{high}}\right|$$

In the formula, $y\pm U_{p}$ is the endpoint value of the coverage interval of the output obtained by GUM in probability p. $y_{\mathrm{low}}$ and $y_{\mathrm{high}}$ are endpoint values of the probability symmetric coverage interval obtained by MCM. Take δ as the numerical tolerance of the standard uncertainty u(y). If $d_{\mathrm{low}}<\delta$ and $d_{\mathrm{high}}<\delta$, the GUM passes the verification, otherwise it fails.

## 4. Calibration Test and the Evaluation Results

### 4.1. Calibration Test System

**Figure 3:**
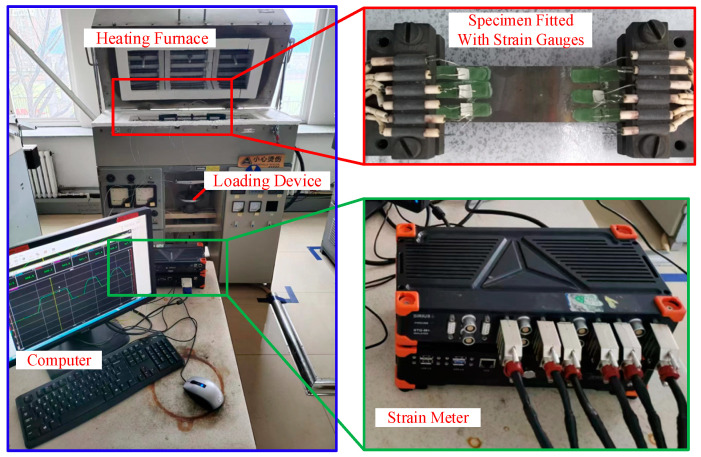
Schematic diagram of test system.

In the calibration test, the specimen is made of DZ125L, a nickel-based superalloy material commonly used in the hot-end parts of aircraft engines. The size of the specimen is 400 mm × 30 mm × 6 mm (length × width × thickness). The high-temperature strain gauge is made of Pt-W alloy grid wire containing 9% W. The grid size of the strain gauge is 3.18 mm × 3.18 mm, and the nominal resistance is 90 Ω. In order to reduce the influence of environmental errors and obtain as many measurement samples as possible, six strain gauges are installed along the axial direction on the upper surface of the specimen. The gauge factor test device is shown in Figure 3. The system consists of a high-temperature heating furnace, temperature controller, loading device, strain gauges, thermocouples, specimen, resistance strain meter, dial indicator, and related computer.

The armored thermocouple of the specification is Φ1 mm, type K was used for temperature measurement, and the measurement accuracy is ±1.5 °C. The thermocouple was installed near the strain gauge and the temperature signal was fed into the DEWESOFT-STGM data acquisition (DEWESOFT Co., Kumberg, Austria) instrument through the DSI-TH-K adapter (DEWESOFT Co., Kumberg, Austria) to realize the simultaneous measurement of temperature and strain. From 25 °C up to 900 °C, the selection of test nodes needs to take into account the capacity of the equipment and the test period, so the equal interval selection of 100 °C was carried out. The GF calibration points were set as 25 °C, 200 °C, 300 °C, 400 °C, 500 °C, 600 °C, 700 °C, 800 °C, and 900 °C. After reaching a constant temperature point, the constant temperature was maintained for more than 15 min to ensure a uniform temperature between the measurement points. The force was loaded through the combination of weights and mechanisms for raising and lowering weights. The loading was stopped when the surface strain of the specimen reached 1000 ± 50 με, and then the test data were recorded before unloading. The Keyence GT2-P12 displacement meter (Keyence Co., Shanghai, China) was selected for deflection f measured, the test accuracy is ±1 μm, and the current signal was connected to the DEWESOFT-STGM data acquisition instrument. The temperature was kept constant, and the loading and unloading operations were repeated three times.

### 4.2. Test Results

**Figure 4:**
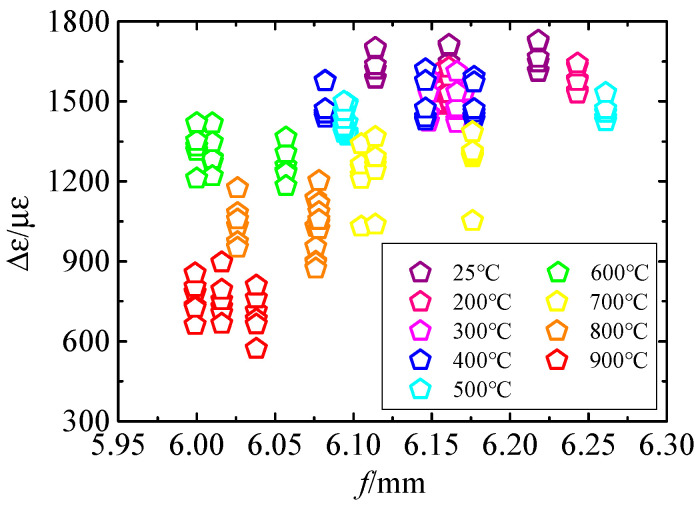
The relationship between the measured value of the strain gauge and the deflection.

In the calibration test, six strain gauges were installed on the upper surface of the specimen. The load was applied three times at each calibration temperature point to obtain three different deflections f. Each deflection value corresponds to six measured values of Δε. The measurement results are shown in Figure 4. At different temperatures, the specific data of strain measurement for loading and unloading are shown in Table A1. The uncertainties of h, l, and a are determined by the maximum allowable error of the measuring instrument. Based on the real data from multiple measurements, the uncertainty is evaluated according to the mathematical model.

**Figure 5:**
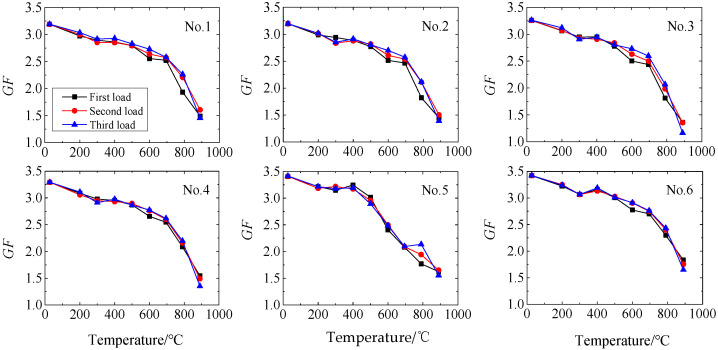
*GF* of strain gauges at different temperatures.

Based on the experimental results, the gauge factor GF of the six strain gauges at different temperatures are calculated, respectively. The results are shown in Figure 5, which shows that GF gradually decreases with the increase in temperature. When the temperature is 25 °C, GF is approximately 3.29, and when the temperature rises to 900 °C, GF decreases to 1.6. When Pt-W is used as the sensitive grid wire material, GF decreases with the increase in temperature. The experimental results are in accordance with this basic law.

### 4.3. Evaluation Results of GUM

The uncertainty analysis of GF at different temperatures is conducted using GUM. The measurement results can be expressed as GF±U, where GF is the estimated value, and U is the half width of the coverage interval of GF at 95% probability. The uncertainty of each uncertainty source and the combined uncertainty at different temperatures can be calculated from the formulas in Section 3.1. The results are shown in Table 1.

**Figure 6:**
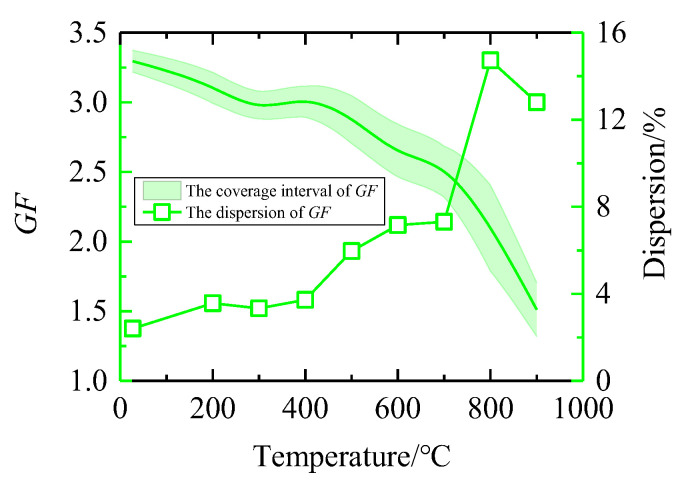
Uncertainty interval and dispersion of GF obtained by GUM.

According to the calculation steps in Section 3.1, the estimated values and uncertainty intervals of GF can be obtained at different temperatures in the range from 25 °C to 900 °C, as shown in Figure 6. It can be seen from the figure that the estimated value of GF decreases with the increase in temperature, which is consistent with the trend of each strain gauge. The uncertainty interval is narrow, and the dispersion at 800 °C is 49.3% higher than that at 700 °C, and the interval variation is unstable. Therefore, MCM analysis is required, and GUM results should be verified.

### 4.4. Evaluation Results of MCM

**Figure 7:**
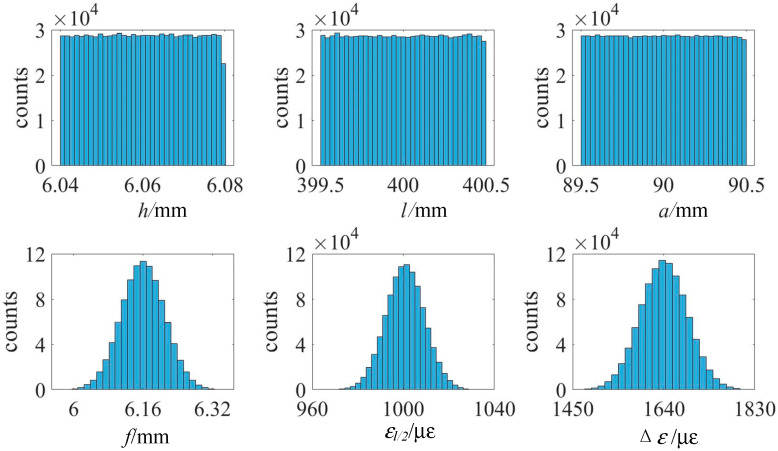
Frequency distribution histogram of variables for MCM.

Based on the test data, MCM is used to simulate and analyze the test results in MATLAB software. At 25 °C, frequency distribution histograms of random numbers such as h, l, a, f, and Δε are shown in Figure 7, and the number of samples is 10^6^. In a high-temperature environment, each input is affected by a variety of random factors such as temperature and is assumed to be the Gaussian distribution.

**Figure 8:**
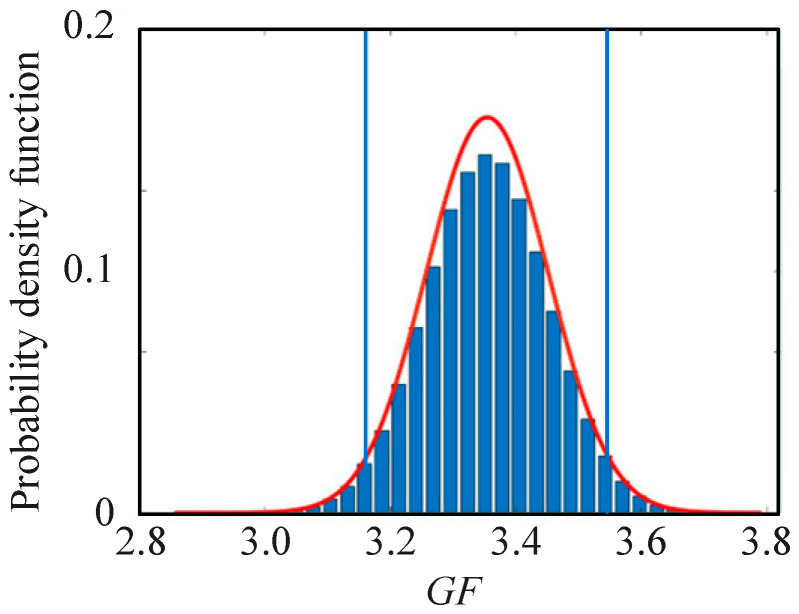
The GF probability distribution by MCM calculates.

The distribution function, estimated value, and standard uncertainty u(GF) of GF are calculated through the mathematical model. The coverage interval at 25 °C is obtained, which is [3.09, 3.49]. Figure 8 shows the frequency distribution histogram and PDF curve of output GF. The two vertical lines indicate the endpoint values of the coverage interval at 95% probability.

**Figure 9:**
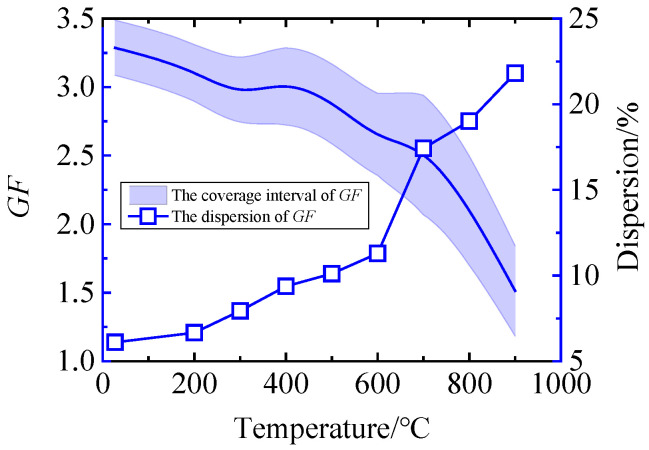
Uncertainty interval and dispersion of GF obtained by MCM.

Based on the same MCM program, the GF of different temperature nodes in the range of 25 °C to 900 °C is calculated. The relationship between the estimated value and its uncertainty coverage interval with temperature variation, as well as the relationship between GF dispersion and temperature are obtained, as shown in Figure 9. Through simulation experiments, large-scale samples can be obtained from MCM, so that the obtained coverage interval is more stable and has less fluctuation. The figure shows that the dispersion of GF gradually increases with the increase in temperature. The dispersion of GF is 6.1% at 25 °C. When the temperature rises to 900 °C, the dispersion of GF increases to 21.8%. Since the material properties vary with temperature, the general trend of dispersion should increase with increasing temperature, and the MCM evaluation results are consistent with this fact.

### 4.5. Comparison Between GUM and MCM

**Figure 10:**
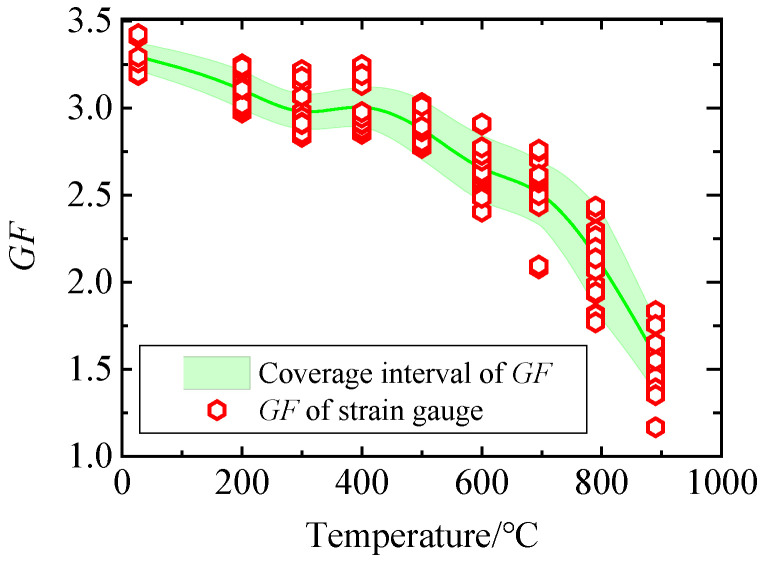
Uncertainty interval obtained by GUM and GF of each strain gauge.

**Figure 11:**
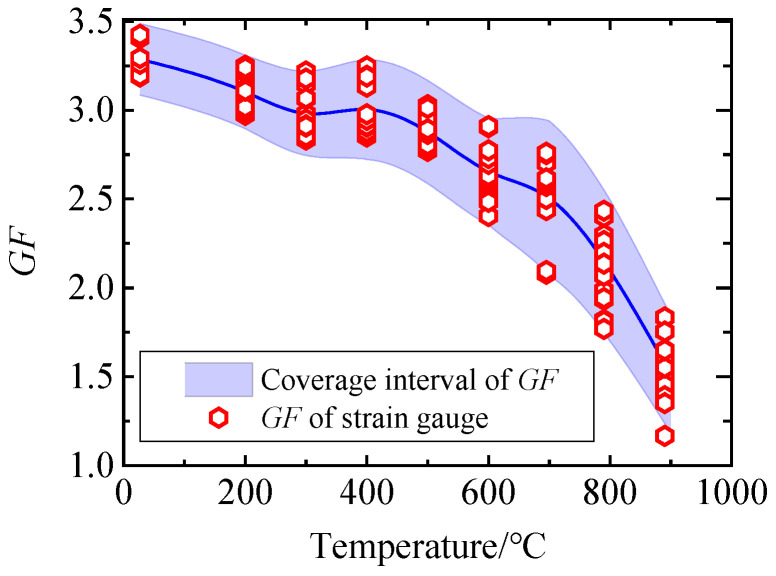
Uncertainty interval obtained by MCM and GF of each strain gauge.

In the field of measurement, GUM and MCM are the mainstream methods for evaluating measurement uncertainty. MCM is based on probability and statistics theory, and its application scope is wider and its credibility is higher. In order to find a more suitable method for evaluating uncertainty, the analysis results of GUM and MCM are compared. Figure 10 and Figure 11 show the relationship between the uncertainty intervals obtained by GUM and MCM and the GF of each strain gauge measured experimentally (Figure 5). It can be seen from Figure 10 that at the specified calibration point, the GF uncertainty coverage interval obtained by GUM cannot contain all the experimental results, and the distortion is large. It can be seen from Figure 11 that at all calibration points, the GF uncertainty coverage interval obtained by MCM can contain the GF values obtained from the experiments and will not be too large. The uncertainty evaluation result of MCM is closer to the real situation, and the reliability is higher. MCM is superior to the GUM, which only uses prior information for uncertainty evaluation.

**Figure 12:**
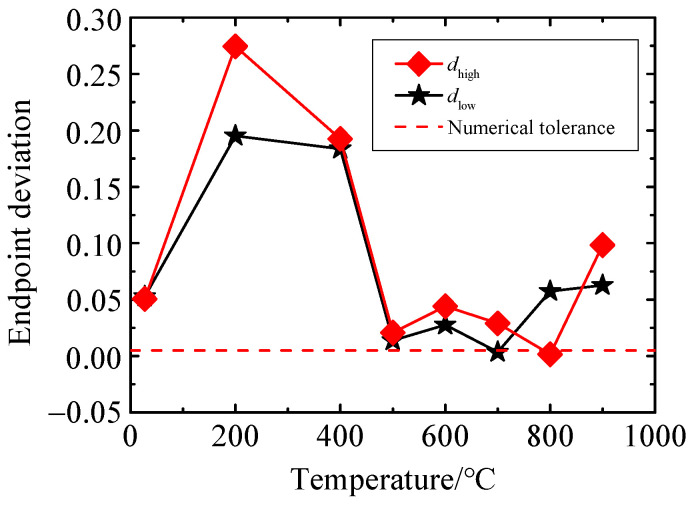
Endpoint deviation value of the interval.

To further verify the reliability of the GUM evaluation results, the numerical tolerance δ verification is carried out using MCM. According to the above analysis, the uncertainty of GF is about 6.1% at 25 °C. When one significant digit is taken, that is, $U(GF)\approx 6\times 10^{-2}$, then the value of δ is $\delta =\frac{1}{2}\times 10^{-2}=0.005$. The $d_{\mathrm{low}}$ and $d_{\mathrm{high}}$ at different temperatures can be calculated from Equations (12) and (13), as shown in Figure 12. The figure shows that GUM fails to pass the verification at different calibration temperatures, and GUM is not suitable for the uncertainty analysis of GF. MCM has no nonlinear restrictions on the model and is not affected by the correlation of input quantities and the complexity of the model. Therefore, MCM can overcome the defects and shortcomings of GUM in evaluating complex models and obtain more reliable measurement uncertainty evaluation.

According to the above comparison, MCM is more suitable for the uncertainty evaluation of high-temperature strain gauges. In order to supplement the deficiency of MCM and find the main source of uncertainty quantitatively, further research is needed.

## 5. Analysis of Uncertain Sources

The calibration test of GF is affected by various factors, including the manufacturing and installation errors of the strain gauges and specimen, the equipment testing error, the nonlinear changes in material properties with temperature, and the temperature error. These errors are reflected in the mathematical model Equation (3) as follows: error of measuring strain with strain meter, error of measuring h, l, a, deflection error of measuring with micrometer, error of measuring temperature with thermocouple, etc. From the above analysis, GUM is not suitable for evaluating the uncertainty of GF calibration tests. The calculation of the major uncertain source is not involved in the evaluation process of MCM. In order to quantitatively evaluate the major uncertain source, the following analysis is carried out.

### 5.1. The Influence of $\frac{\partial GF}{\partial x}$ on the GF

According to the calculus theory, the influence of various factors on GF is related to the derivative of the mathematical model Equation (3) with respect to a single variable. Let $\frac{\partial GF_{i}}{\partial h}$ be defined as the sensitivity. The relationship between sensitivity $\frac{\partial GF_{i}}{\partial h}$ and GF can be calculated from Equation (12):

(12) $$\frac{\partial GF_{i}}{\partial h}=\frac{2\Delta \varepsilon_{i}}{12\times f_{l/2}}\times (3l^{2}-4a^{2})\times (-\frac{1}{h^{2}})=GF_{i}\times (-\frac{1}{h})$$

Similarly, the sensitivity of GF to other design variables is as follows:

(13) $$\frac{\partial GF_{i}}{\partial l}=\frac{2\Delta \varepsilon_{i}}{12\times h\times f_{l/2}}\times 6l=GF_{i}\times \frac{6l}{3l^{2}-4a^{2}}$$

(14) $$\frac{\partial GF_{i}}{\partial a}=\frac{2\Delta \varepsilon_{i}}{12\times h\times f_{l/2}}\times (-8a)=GF_{i}\times \frac{-8a}{3l^{2}-4a^{2}}$$

(15) $$\frac{\partial GF_{i}}{\partial f_{l/2}}=\frac{2\Delta \varepsilon_{i}}{12\times h}\times (3l^{2}-4a^{2})\times (-\frac{1}{f_{l/2}^{2}})=GF_{i}\times \frac{-1}{f_{l/2}}$$

(16) $$\frac{\partial GF_{i}}{\partial \Delta \varepsilon }=\frac{2}{12\times h\times f_{l/2}}\times (3l^{2}-4a^{2})=GF_{i}\times \frac{1}{\Delta \varepsilon }$$

**Figure 13:**
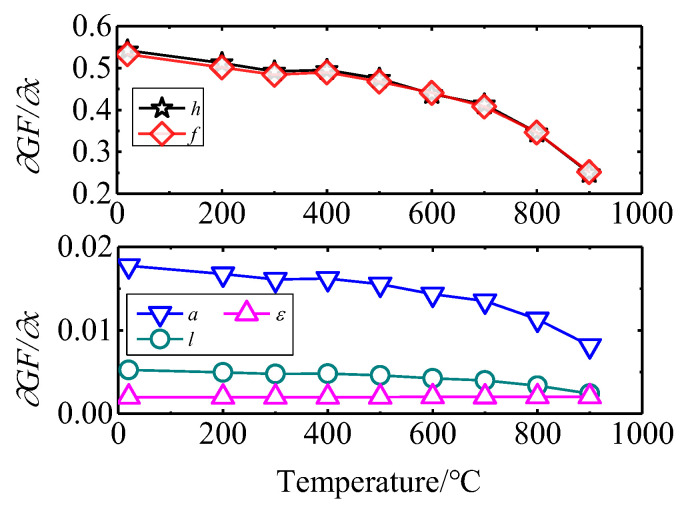
The sensitivity of GF to the design variables.

The dependence of sensitivity on temperature is shown in Figure 13. It can be seen that $\frac{\partial GF_{i}}{\partial h}$ and $\frac{\partial GF_{i}}{\partial f_{l/2}}$ are large and decrease as the temperature increases. $\frac{\partial GF_{i}}{\partial h}$ is about 0.54 at 25 °C and is reduced to 0.25 when the temperature reaches 900 °C. However, $\frac{\partial GF_{i}}{\partial l}$, $\frac{\partial GF_{i}}{\partial a}$, and $\frac{\partial GF_{i}}{\partial \Delta \varepsilon }$ are very small; they are all less than 0.02. Therefore, l, a, and Δε have little influence on GF, but GF is sensitive to h and $f_{l/2}$.

### 5.2. The Influence of the Uncertainty of the Input u(x) on U(GF)

The uncertainty of each input has a range. l and a are measured by meter ruler, the uncertainty is [±0.05, ±0.5] mm. h is measured by vernier calipers, and the uncertainty is [±0.02, ±0.05] mm. $f_{l/2}$ is measured using a dial indicator with an uncertainty of [0.004, 0.018] mm. Δε is measured by strain meter, with an uncertainty of [15, 75] με. As the temperature rises, the measurement uncertainty will be affected by temperature and thermal expansion coefficient, etc.

**Figure 14:**
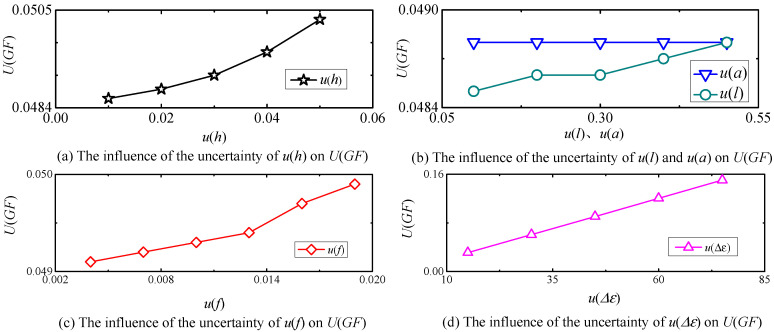
The influence of the uncertainty of each factor on U(GF).

The relationship between the uncertainty u(x) of each input and the uncertainty U(GF) of GF is obtained by the MCM program, as shown in Figure 14. It can be seen from Figure 14a that U(GF) increases with the increase in u(h), but the increased effect is not significant, and U(GF) increases from 0.0486 to 0.0503. As shown in Figure 14b, the increases in u(l) in its error range results in a weak increase in U(GF) in the range of 0.0485~0.0488. The uncertainty of u(a) has no effect on U(GF). As can be seen from Figure 14c, with the increase in u(f), the increase in U(GF) is not significant, increasing from 0.0486 to 0.0494. Figure 14d indicates that U(GF) increases linearly with the increase in u(Δε). When u(Δε) is 15 με, U(GF) is 0.0312, and when u(Δε) reaches 75 με, U(GF) reaches 0.1503. Therefore, u(Δε) has the greatest influence on U(GF).

### 5.3. The Influence of the PDF of Each Factor on GF

From the previous analysis, it can be assumed that Δε is the major uncertain source in the evaluation of the gauge factor GF. Therefore, it is expected that the PDF of GF will also change significantly for different PDFs of Δε. To prove this fact, different PDFs are assigned to the input Δε and their effect on the PDF of the output GF is studied.

**Figure 15:**
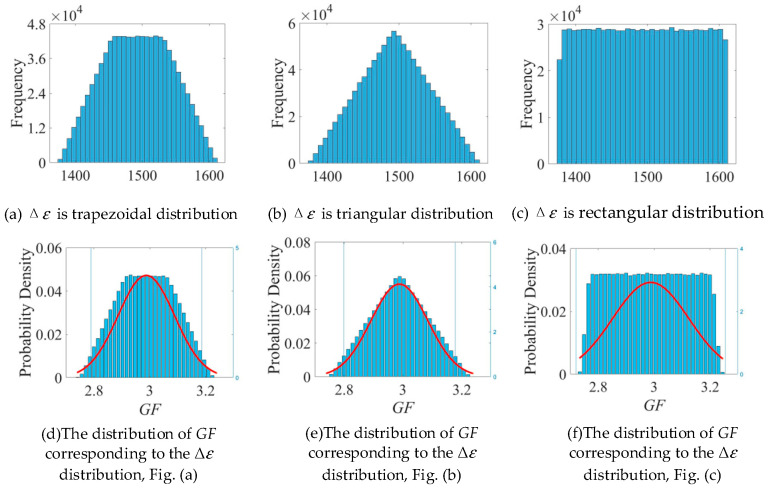
The influence of distribution of Δε on distribution of GF.

**Figure 16:**
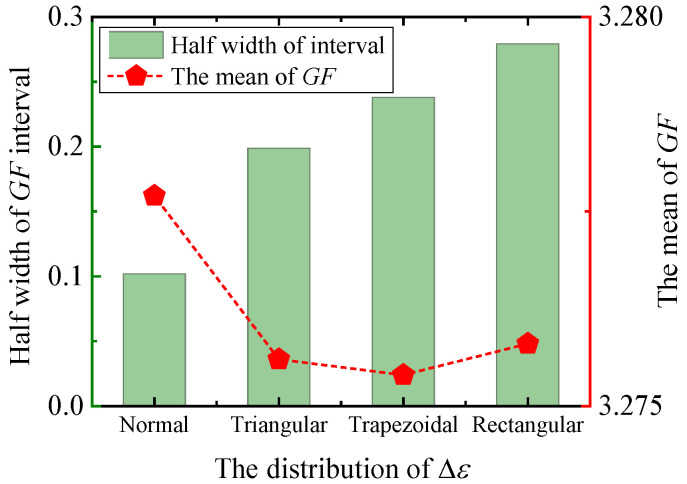
The influence of the distribution of Δε on the uncertainty of GF.

Figure 15 shows the assumed Δε distribution and the corresponding frequency distribution of the output GF. As can be seen from the figure, the distribution of GF is strongly correlated with the distribution of Δε. Firstly, Δε is assumed to be trapezoidal distribution (Figure 15a), which leads to the output GF tending to be a trapezoidal distribution. The mean value is 3.28, which is little changed from the normal distribution. The uncertainty interval is wider, and the uncertainty is about 0.24. In addition, the cases where Δε is triangular distribution and rectangular distribution are analyzed, and the analysis results are shown in Figure 16. When the means of the input quantities are the same, the distribution function of Δε has little influence on the mean of GF, in the range of 3.275~3.280. When Δε is normally distributed, U(GF)=0.10, which is the smallest value, and the uncertainty interval is the narrowest. When Δε is a rectangular distribution, the uncertainty interval is the widest, which is 0.28. For convenience of calculation, let $k_{\mathrm{PDF}}=1$ when Δε is normally distributed. When Δε is trapezoidal distribution, let $k_{\mathrm{PDF}}=2.34$. When Δε is triangular, $k_{\mathrm{PDF}}=1.95$. Let $k_{\mathrm{PDF}}=2.74$ when Δε is uniformly distributed.

Through the above analysis, the derivative of the mathematical model with respect to each input, the uncertainty u(x) of each input, and the PDF of each input all affect the uncertainty U(GF). Therefore, the concept of the weight coefficient W is proposed as the criterion to judge the major uncertain source. The weight coefficient W is expressed by Formula (17):

(17) $$W=\frac{\partial GF}{\partial h}\times u\times k_{\mathrm{PDF}}$$

**Figure 17:**
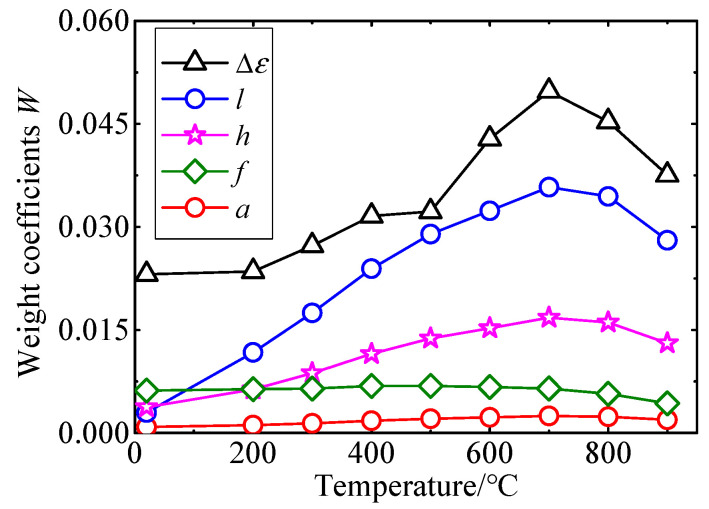
Weight coefficients of each input at different temperatures.

At different temperatures, the weight coefficients W of each input is calculated by Equation (17), and the results are shown in Figure 17. As can be seen from the figure, the weight coefficients of Δε and l are larger, while the W values of the other three input quantities are smaller. This is particularly prominent when the temperature is higher than 300 °C. Therefore, Δε is the major uncertain source.

## 6. Conclusions

In this paper, the calibration test of high-temperature strain gauge GF was carried out, and the uncertainty of the test results was evaluated by GUM and MCM, respectively. The influence of each input on the uncertainty of GF was studied, and the concept of weight coefficient W was proposed. The conclusions are as follows:

(1) The calibration test results of the high-temperature strain gauge show that the GF decreases with the increase in temperature nonlinearly. At 25 °C, GF is about 3.29, and when the temperature reaches 900 °C, GF decreases to 1.6.
(2) GUM and MCM are used to evaluate the uncertainty of the calibration test, and the results are compared and analyzed. The uncertainty obtained by GUM is too small, but it cannot cover all the test results and cannot accurately characterize the true dispersion of GF. The endpoint deviation values are all greater than the numerical tolerance, that is, GUM has not passed the verification and is not suitable for the uncertainty evaluation of the strain gauge GF.
(3) MCM can obtain more samples through large-scale stochastic numerical simulation. The evaluation results can be better matched with the test results, and the evaluation results are more accurate and effective. The dispersion is 6.1% at 25 °C, and it reaches 21.8% when the temperature rises to 900 °C.
(4) The concept of weight coefficient W is proposed, which includes the sensitivity of the mathematical model to each input, the uncertainty u(x) of each input, and the PDF of each input. The analysis proves the influence of the three aspects on U(GF), which can be an effective supplement to MCM, and the major uncertain source Δε in the mathematical model can be quantitatively analyzed. The uncertainty evaluation can be applied to other fields and provides important information for improving the stability of the installation process.

Although this study has made some achievements, it is still limited to the post-processing of the experimental results. It is expected that the stochastic finite element method will be used to analyze the structural uncertainty theoretically in the future.

## Figures and Tables

**Table 1:** The uncertainty of each uncertain source.

	*T*/°C	25	200	300	400	500	600	700	800	900
Uncertainty										
u(h)/mm		0.01	0.02	0.03	0.04	0.05	0.06	0.07	0.08	0.09
u(l)/mm		0.29	1.21	1.88	2.55	3.23	3.91	4.59	5.26	5.94
u(a)/mm		0.29	0.39	0.51	0.64	0.78	0.92	1.07	1.22	1.37
u(Δf)/mm		0.052	0.049	0.012	0.048	0.096	0.030	0.039	0.029	0.020
u(Δε)/με		3.23	27.22	54.58	30.88	28.33	38.32	13.35	31.54	28.82

## Data Availability

Data are contained within the article.

## Appendix A

**Table A1:** The strain measurement results for loading and unloading at different temperatures.

T/°C	First Load: Deflection/Strain (mm/με)						Second Load: Deflection/Strain (mm/με)						Third Load: Deflection/Strain (mm/με)					
25	6.161						6.218						6.114					
	1596	1599	1630	1647	1705	1712	1611	1613	1647	1664	1722	1728	1585	1587	1620	1636	1696	1701
200	6.156						6.161						6.243					
	1487	1494	1533	1544	1608	1612	1502	1508	1537	1531	1594	1627	1539	1530	1582	1577	1631	1643
300	6.171						6.166						6.148					
	1451	1473	1478	1494	1577	1537	1430	1420	1468	1477	1612	1535	1456	1425	1452	1454	1587	1532
400	6.146						6.177						6.082					
	1428	1441	1473	1474	1621	1577	1431	1444	1459	1471	1592	1572	1447	1439	1455	1471	1578	1576
500	6.096						6.094						6.261					
	1385	1372	1377	1421	1493	1488	1383	1394	1405	1434	1464	1499	1438	1426	1426	1458	1471	1532
600	6.057						6.010						6.000					
	1258	1237	1232	1307	1183	1365	1288	1273	1284	1348	1220	1418	1330	1315	1331	1352	1211	1419
700	6.105						6.114						6.176					
	1251	1223	1209	1263	1031	1340	1278	1261	1242	1291	1038	1367	1294	1291	1303	1313	1051	1385
800	6.076						6.026						6.078					
	954	898	895	1030	873	1135	1081	1033	972	1057	951	1175	1118	1044	1021	1085	1055	1202
900	6.016						5.999						6.038					
	728	707	666	754	793	896	783	732	661	726	804	855	714	682	572	663	761	809
